# HNRNPA2B1, as a m^6^A Reader, Promotes Tumorigenesis and Metastasis of Oral Squamous Cell Carcinoma

**DOI:** 10.3389/fonc.2021.716921

**Published:** 2021-09-23

**Authors:** Feiya Zhu, Tianru Yang, Mianfeng Yao, Ting Shen, Changyun Fang

**Affiliations:** ^1^ Department of Prosthodontics, Center of Stomatology, Xiangya Hospital, Central South University, Changsha, China; ^2^ Research Center of Oral and Maxillofacial Tumor, Xiangya Hospital, Central South University, Changsha, China; ^3^ Institute of Oral Cancer and Precancerous Lesions, Central South University, Changsha, China

**Keywords:** Oral squamous cell carcinoma, HNRNPA2B1, N6-methyladenosine, EMT, Metastasis

## Abstract

N6-methyladenosine (m^6^A) modification is the most prevalent modification on eukaryotic RNA, and the m^6^A modification regulators were involved in the progression of various cancers. However, the functions of m^6^A regulators in oral squamous cell carcinoma (OSCC) remain poorly understood. In this study, we demonstrated that 13 of 19 m^6^A-related genes in OSCC tissues are dysregulated, and HNRNPA2B1 was the most prognostically important locus of the 19 m^6^A regulatory genes in OSCC. Moreover, HNRNPA2B1 expression is elevated in OSCC, and a high level of HNRNPA2B1 is significantly associated with poor overall survival in OSCC patients. Functional studies, combined with further analysis of the correlation between the expression of HNRNPA2B1 and the EMT-related markers from the TCGA database, reveal that silencing HNRNPA2B1 suppresses the proliferation, migration, and invasion of OSCC *via* EMT. Collectively, our work shows that HNRNPA2B1 may have the potential to promote carcinogenesis of OSCC by targeting EMT *via* the LINE-1/TGF-β1/Smad2/Slug signaling pathway and provide insight into the critical roles of HNRNPA2B1 in OSCC.

## Introduction

Oral squamous cell carcinoma (OSCC) is a major type of oral cancer which caused an estimated 177,384 deaths in 2018 (1). The incidence of OSCC has increased in recent years, especially in young adults (<40 years old) (2). Despite the great progress of therapy in the last three decades, the survival of patients with OSCC has not changed significantly (3, 4), encouraging clinicians and researchers to look for promising molecular targets or factors that might modify the disease outcome.

Recent studies have shown that chemical modifications on RNA contribute to gene expression control that is linked to human cancers (5). N6-Methyladenosine (m^6^A) is one of the most abundant modifications in eukaryotic messenger RNAs (mRNAs) (6). More than 10,000 m^6^A peaks have been identified on over 25% of transcripts, with an average of one to three modifications per transcript (6–8). The m^6^A modification is dynamic and reversible in mammalian cells, which could be installed by methyltransferases (writers) consisting of METTL3 (methyltransferase-like 3), METTL14, WTAP (Wilms tumor 1-associated protein), KIAA1429, RBM15 (RNA-binding motif protein 15), and its paralog (RBM15B), whereas its removal is regulated by demethylases (erasers) such as FTO (fat mass and obesity-associated) and ALKBH5 (ALKB homolog 5). In addition, the specific RNA-binding proteins (readers), including the YT521-B homology (YTH) domain family and certain members of the heterogeneous nuclear ribonucleoprotein (HNRNP) family, could recognize and bind to the m^6^A motif, regulating RNA metabolism including RNA stability, degradation, pre-mRNA splicing, transport, nuclear export, localization, translation, and other processes (9, 10). Abnormal m^6^A modifications regulate the expression of target genes acting as proto-oncogene or tumor suppressor in the initiation and progression of a wide range of tumors (9, 11), including acute myeloid leukemia (AML) (12, 13), lung squamous cell carcinoma (LSCC) (14, 15), glioblastoma stem-like cell (GSC) (16, 17), human hepatocellular carcinoma (HHC) (18–20), breast cancer (BC) (21, 22), and ovarian cancer (OC) (23, 24). Accumulating evidence has shown that the m^6^A components METTL3 and ALKBH5 are closely related with OSCC tumorigenesis, metastasis (25, 26), and cisplatin resistance (27) in the last few years. Heterogeneous nuclear ribonucleoproteins A2B1 (HNRNPA2B1), one of the members of the HNRNP family, is a pre-mRNA-binding protein that participates in mRNA subcellular localization, stability, and translation (28). Additionally, elevated HNRNPA2B1 level was reported in various tumors (29–31). It is said that HNRNPA2B1 promotes the progression of esophageal cancer by upregulating ACLY and ACC1 (30). However, whether HNRNPA2B1 could promote the development of OSCC is not yet clear.

In this study, we systematically analyzed the expression of 19 m^6^A-related genes using The Cancer Genome Atlas (TCGA) dataset, as well as the prognostic role of m^6^A regulators in OSCC through univariate Cox regression analysis and LASSO Cox regression model. The results show that a four-gene prognostic signature including METTL3, YTHDF3, HNRNPC, and HNRNPA2B1 could predict overall survival of OSCC patients. After a comprehensive analysis, we found that HNRNPA2AB1 is an independent prognostic risk factor for OSCC. Combining with the clinical data from our hospital and *in vitro* functional studies, we found that HNRNPA2B1 may act as an oncogenic role in OSCC progression. Our previous study reported that LINE-1 might regulate the OSCC proliferation, migration, and invasion through epithelial and mesenchymal transition (EMT). Here, in the current study, we found that HNRNPA2B1 may mediate the EMT process by regulating LINE-1 expression, thereby promoting the proliferation and metastasis of OSCC cells, indicating that HNRNPA2B1 may be a novel factor with the potential to regulate the proliferation and migration of OSCC cells by targeting EMT *via* the LINE-1/TGF-β1/Smad2/Slug signaling pathway.

## Materials and Methods

### Patient Tumor Sections

The tumor samples (primary OSCC tissue specimens and adjacent matched normal mucosa tissues) were obtained from 38 OSCC patients with or without lymph node metastasis who underwent surgery at the Department of Oral and Maxillofacial Surgery of the Second Xiangya Hospital. These patients underwent surgery between April 2011 and July 2013, and the follow-up period used for survival analyses ended in May 2018. No patients involved in this investigation received chemotherapy before surgery. Tumor histology and grading were classified according to the WHO guidelines. The details of the patients used in this study are present in **Table 1**.

**Table 1:** Statistical analyses of factors associated with survival in OSCC patients with the multivariate Cox proportional hazards models.

Variables	HR	HR.95L	HR.95H	*p*
Age (years)	1.395	0.911	2.137	0.125
≥60 *vs.*<60				
Gender	0.982	0.637	1.514	0.935
Male *vs.* female				
Grade	1.681	1.027	2.750	0.038
Poor *vs.* well-moderate				*
Stage	1.485	0.563	3.914	0.423
Stage (III + IV) *vs.* stage (I + II)				
T classification	2.104	1.165	3.800	0.013
T (3 + 4) *vs.* T (1 + 2)				*
Metastasis	1.441	0.891	2.329	0.136
Positive *vs.* negative				
HNRNPA2B1 expression	2.054	1.33	3.152	<0.001
Positive *vs.* negative				***

* and *** indicate p < 0.05 and p < 0.001, respectively.

### Immunohistochemical Staining and Analysis

Immunohistochemistry (IHC) was performed to evaluate HNRNPA2B1 and LINE-1 expression using the standard protocol. The tissue sections were dewaxed with xylenes, hydrated with graded alcohols. Endogenous peroxidase was inhibited with 3% hydrogen peroxide. Antigen retrieval was performed using citrate buffer (pH 6.0) in a microwave oven. Anti-rabbit HNRNPA2B1 (1:50, ProteinTech, USA) and anti-mouse ORF1p (1:500, Sigma-Aldrich, Germany) primary antibodies were used to the sections overnight at 4°C, then incubated with a secondary antibody for an hour at room temperature. Signals were visualized by using a 3,30-diaminobenzidine (DAB) detection kit (DakoCytomation, Denmark). Sections were counterstained with hematoxylin, dehydrated, and mounted. The expressions of HNRNPA2B1 and ORF-1p are scored by two researchers. For each specimen, one score was assigned according to the percentage of positive cells: <25%: one point; 26%–50%: two points: 51%–75%, three points; and 76%–100%: four points. A second score was assigned according to the intensity of the staining, with negative staining equaling 0, weak staining equaling one point, moderate staining equaling two points, and strong staining equaling three points. IHC score was then calculated by multiplying the two scores described above. If the expression score was ≥5, the tissue was considered as positive expression.

### m^6^A Component Transcriptome Profiling Obtaining and Differential Analysis

The transcriptome profiles of 330 OSCC and 32 normal cases were downloaded from TCGA (https://tcga-data.nci.nih.gov/tcga/). TCGA is a publicly available dataset. No ethics approval was needed. Based on previous literatures, we have identified 19 m^6^A-related genes, including METTL3, METTL14, METTL16, WTAP, KIAA1429, RBM15, FTO, ALKBH5, ZC3H13, YTHDF1, YTHDF2, YTHDF3, YTHDC1, YTHDC2, HNRNPC, HNRNPA2B1, LRPPRC, IGF2BP1, and FMR1. Differential expression analysis was performed in R software, with the cutoff value of |log2(fold change [FC]|>2 and *p <*0.05. The heatmap and boxplot were constructed using the ggplot2 package in R software.

### Cluster Analysis and Principal Component Analysis

Cluster analysis was performed according to the m^6^A-related gene transcriptome profiles. Meanwhile, the result of cluster analysis was used to make a principal component analysis (PCA). The patient’s clinical data of survival time and survival status were extracted from 330 OSCC samples. The correlation analysis between clinical features and clustering results was performed through R. Finally, heatmaps were constructed through “pheatmap,” survival, and “survminer” packages.

### Survival Analysis

In this study, we performed three survival analyses. For the first analysis, we divided all OSCC patients from the TCGA database into cluster 1 and cluster 2 based on the results of cluster analysis. For the second analysis, we divided OSCC patients into high-risk groups and low-risk groups according to the Cox regression model. For the third analysis, we divided the OSCC patient samples from the department or TCGA database into positive expression and negative expression, or high expression (with counts higher than the median) and low expression (with counts lower than the median). Then, we used the Kaplan–Meier method to analyze the candidate genes of significant prognostic value with *p* < 0.05.

### Cox Risk Regression Establishment

First, the original expression profile data of m^6^A-related genes were normalized by log2(x + 1), and prognosis-associated factors were selected by univariate Cox regression. Next, we performed Cox regression analysis combined with LASSO regression to establish a risk regression and the penalty regularization parameter lambda (λ) was chosen through the cross-validation routine with an n-fold equal to 10 by using R package “glmnet” (32). Meanwhile, “lambda.min” was identified to pick out the variables. Finally, HNRNPA2B1, METTL3, YTHDF3, and HNRNPC were enrolled in risk Cox regression and survival analysis. Moreover, univariate and multivariate Cox regressions are performed through survival and “forestplot” packages to identify the independent prognostic factor of OSCC. Moreover, we evaluate the reliability of the risk regression through “survivalROC” package in R software.

### Machine Learning

To identify the key gene of m^6^A, we used the caret package to construct three different machine learning models to investigate features of importance involving 19 m^6^A-related genes. Neural network (nn), random forest (rf), and gradient boosting machine (gbm) were used to rank the 19 genes by their expression levels in two disease states (tumor and normal). The most important feature of the three models was selected as the key gene; results were visualized by ggplot2 package.

### EMT Scores and Gene Set Enrichment Analysis

To provide a quantitative estimate of EMT, we constructed a model by multinominal logistic regression; each sample is given EMT scores, which range from 0 (pure epithelial) to 2 (pure mesenchymal), with a score of 1 indicating a maximal hybrid epithelial/mesenchymal (E/M) phenotype (33). The transcriptome profiles of OSCC used for GSEA were downloaded from TCGA (https://tcga-data.nci.nih.gov/tcga/) and GEO data (https://www.ncbi.nlm.nih.gov/geo/query/acc.cgi?acc=GSE138206). GSEA (34, 35) was performed with KEGG gene set (c2) or oncogenic signature gene set (c6) collections of the Molecular Signature Database v7.0(http://www.gsea-msigdb.org/gsea/downloads.jsp).

### Cell Culture, Antibodies, and Reagent

CAL27 and SCC4 cells were cultured in Dulbecco’s modified Eagle medium (DMEM) supplemented with 10% fetal bovine serum (Invitrogen, Germany), 2 mM L-glutamine (Invitrogen, Germany), and antibiotics (50 μg/ml penicillin–streptomycin, Invitrogen, Germany). Mouse anti-α-tubulin, rabbit anti-E-cadherin (24E10), and anti-N-cadherin (D4R1H) were from Cell Signaling Technology (Beverly, MA). Rabbit anti-Snail and anti-TGF-β1 were from Abcam (Cambridge, MA). Rabbit anti-HNRNPA2B1 was from ProteinTech (Rosemont, IL, USA), and mouse anti-ORF1p was purchased from Sigma-Aldrich (clone 4H1, Germany).

### RNA Interference

Commercial shRNA plasmid vectors (U6–MCS–Ubiquitin–Cherry–IRES–puromycin) carrying target-specific ENAuences against human HNRNPA2B1 and non-target scrambled control were purchased from GeneChem (Shanghai, China). Lentiviral particles were produced in HEK293T packaging cells with shRNA, psPAX2 (#12260, Addgene), and pMD2.G (#12259, Addgene) plasmids. Then, CAL27 and SCC4 cells were infected with 0.45-μm-pore filtered viral supernatants. Infected cells of CAL27 and SCC4 were selected for 2 weeks using 1 μg/ml puromycin. The expression of HNRNPA2B1 was confirmed by RT-PCR and Western blot. Oligonucleotide sequences for shRNAs are the following: HNRNPA2B1 shRNA#1 and shRNA#2 are 5′-TGACAACTATGGAGGAGGAAA-3′ and 5′-AGAAGCTGTTTGTTGGCGGAA-3′, respectively. The sequence of scrambled shRNA is 5′-TTCTCCGAACGTGTCACGT-3′.

### HNRNPA2B1 Overexpression

The HNRNPA2B1 overexpression lentiviral vector-plasmid (GV208, Ubi-MCS-HNRNPA2B1-EGFP) was purchased from GeneChem (Shanghai, China) and used to transfect the HEK293T packaging cells. CAL27 and SCC4 cells were transfected with lentivirus containing HNRNPA2B1 cDNA for subsequent analysis.

### Quantitative Real-Time Polymerase Chain Reaction

Total RNA was isolated using TRIzol Reagent (Invitrogen, Thermo Fisher Scientific, USA) and reserved transcribed with TaqMan Reverse Transcription Reagents Kit (Applied Biosystems, Foster City CA). The resulting cDNAs (5 μl) were used as templates for qRT-PCR with specific primers including HNRNPA2B1 (forward: 5′-TAGTACTACAGGTTGCTGCC-3′, reverse: 5′-CTGTGAGGCTAGACTAGTAGT-3′) and GAPDH (forward: 5′-ACAACTTTGGTATCGTGGAAGG-3′, reverse: 5′-GCCATCACGCCACAGTTTC-3). qRT-PCR was carried out with Power SYBR Green PCR Master Mix. The qRT-PCR cycling conditions were as follows: 95°C for 10 s; 40 cycles of 95°C for 5 s, 60°C for 20 s, 95°C for 60 s, 55°C for 30 s, and 95°C for 30 s. Fold changes were determined by comparing the ΔCT value of each product normalized to GAPDH as an internal control.

### Western Blot Analysis

The whole-cell lysates were prepared using RIPA lysis buffer according to the standard protocol. For Western blot, 15 μg protein for each group was resolved by SDS-PAGE and transferred onto polyvinyldinefluoride (PVDF) membranes (Millipore, Billerica, MA). The membranes were blocked with appropriate primary antibodies indicated above. After incubating with secondary antibody, the membranes were visualized with enhanced chemiluminescence and the bands were analyzed with ImageJ software.

### Cell Proliferation Assay

Cell proliferation was measured using Cell Counting Kit-8 (CCK8, Bimake, Houston, TX, USA) assay. CAL27 and SCC4 cells were seeded in 96-well plates, three wells for each group, with each well containing 2*10^3^ cells. At 24, 48, 72, 96, and 120 h, 10 μl of CCK8 reagent was added to each well and the cells were incubated for 1 h at 37°C. Then the absorbance at 450 nm was measured using the microplate reader.

### Wound Healing Assay

CAL27 and SCC4 shRNA stable cells were plated in six-well plates at a density of 2.5 × 10^6^ cells/well. After the cells reached 100% confluency, straight wounds were made by using 200-μl tips and the cells were incubated in medium with 1% FBS. The healing of the gap was captured at 0, 24, 48, and 72 h. Experiments were repeated at least three times.

### Cell Invasion Assay

Cell invasion assays were performed in 24-well plates using chambers with 8-μm pores (Corning Incorporated, Kennebunk, ME, USA) coated with an indicator layer of growth factor reduced Matrigel. 5 × 10^4^ CAL27 and SCC4 stable cells were seeded in the upper chamber under serum-free conditions, and DMEM with 10% FBS was placed in the lower chamber. After 24 h, cells on the upper chamber were scraped and washed away; cells on the lower chambers were fixed in 4% formaldehyde and stained with 0.5% crystal violet for 20 min. Next, the invasion cell numbers of each field were counted *via* microscope.

### m^6^A RNA Methylation Assay

TRIzol (Thermo Fisher Scientific, Waltham, MA, USA) was used to extract total RNA from OSCC patients’ tissue samples. m^6^A methylation was quantified according to the guideline of m^6^A RNA Methylation Assay Kit (ab185912, Abcam, Cambridge, MA).

### RNA Immunoprecipitation Assay

RIP was performed with Magna RIP RNA-binding protein immunoprecipitation kits (Millipore, Billerica, MA). Antibodies against HNRNPA2B1 were used. Total RNA from cells was extracted and depleted of ribosomal RNA. The RNA protein complexes were washed and mixed with 900 μl RIP Immunoprecipitation Buffer. Next, RNA was purified with 150 μl proteinase K buffer. Finally, RNAs were extracted and evaluated by qRT-PCR, which was normalized to input and IgG.

The primers of LINE-1 are forward: 5′-GGGCTGCACCTGTCAAGATA-3′, reverse: 5′-ACCTTGCCATCTTTTCCCGT-3′.

### Statistical Analysis

All analyses were performed with the SPSS 25.0 software (SPSS Inc., USA), R software (Version 4.0.3), and GraphPad Prism software version 7.0 (GraphPad Software, Inc., La Jolla, CA, USA). If there are no special instructions, all bioinformatics analysis and visualization in this study are performed using R software. Statistical significance was determined by Student’s t-test. The analysis of overall survival and progression-free survival time was calculated using the Kaplan–Meier method, and the differences in survival between the groups were compared using a log-rank test. The effect of clinicopathological factors on survival was determined with univariate and multivariate Cox proportional hazard models. Correlations between HNRNPA2B1 and EMT marker expression levels were computed using Pearson correlation analysis. Data from three independent experiments were presented as mean ± SD. A p-value of <0.05 was considered statistically significant. *, **, *** and **** indicated *p* < 0.05, *p* < 0.01, *p* < 0.001 and *p* < 0.001, respectively.

## Results

### m^6^A Level and m^6^A Regulatory Genes Were Abnormally Expressed in OSCC

**Figure 1:**
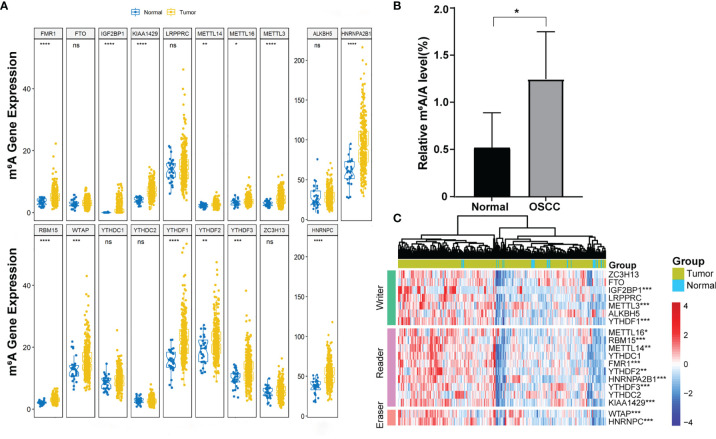
The expression of 19 m^6^A-related genes in OSCC. **(A)** Boxplot visualizing the expression of m^6^A-related genes in 330 OSCC samples and 32 normal samples. **(B)** m^6^A level was detected in six pairs of OSCC tissues and adjacent normal tissues (*p* < 0.05). **(C)** The expression levels of 19 m^6^A-related genes in OSCC were displayed *via* a heatmap. The ascending normalized expression level in the heatmaps is colored from blue to red. *, **, *** and **** indicated p<0.05, p<0.01, p<0.001 and p<0.0001, respectively. “ns” indicates no significance.

More and more evidence showed that m^6^A RNA methylation promotes tumor initiation and progression. However, there are fewer studies to explore the role of m^6^A in OSCC. To study the role of m^6^A regulatory genes in OSCC, we detect the m^6^A level in OSCC and normal adjacent tissues. The result showed that the m^6^A level upregulated in tumor tissues compared with normal adjacent tissues (**Figure 1B**). Further, we evaluated all 19 m^6^A-related genes in 330 OSCC samples and 32 normal samples from the TCGA database, including METTL3, METTL14, METTL16, WTAP, KIAA1429, RBM15, FTO, ALKBH5, ZC3H13, YTHDF1, YTHDF2, YTHDF3, YTHDC1, YTHDC2, HNRNPC, HNRNPA2B1, LRPPRC, IGF2BP1, and FMR1. The expression profiles of these genes were extracted from the transcriptome data, and it was found that 13 genes were abnormally expressed in tumor tissues compared with control (**Figures 1A, C** **)**.

### Consensus Clustering of m^6^A-Related Genes Identified Two Clusters of OSCC With Different Clinical Outcomes

**Figure 2:**
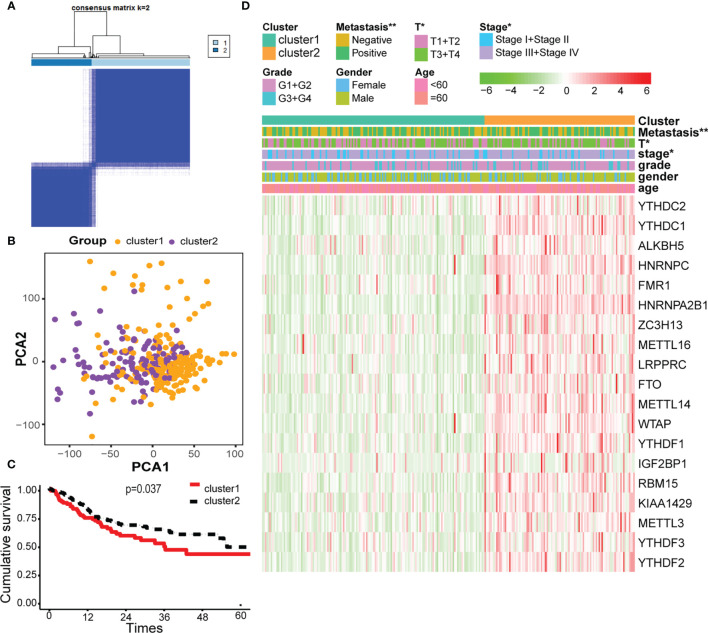
Cluster analysis based on m^6^A-related genes. **(A)** Consensus matrix for the two groups from the TCGA database. **(B)** Principal component analysis (PCA) score plot of the two clusters has few overlaps. **(C)** Overall survival analysis between cluster 1 and cluster 2. **(D)** Heatmap of the 19 m^6^A-related genes, showing distinct expression profiles for correlation of cluster analysis and clinical characteristics. * and ** indicated p<0.05 and p<0.01, respectively.

Cluster analysis was performed to analyze the 330 OSCC samples from the TCGA database. Based on the expression similarity of m^6^A-related genes, k = 2 was demonstrated to be the most appropriated selection to divide the OSCC patient cohort into two clusters, namely, cluster 1 and cluster 2 (**Figures 2A, B** **)**. Subsequently, the overall survival rate of the two clusters estimated by Kaplan–Meier was significantly different (**Figure 2C**). On the basis of correlation analysis of clinical characteristics, an obvious difference was found between cluster 1 and cluster 2 for the metastasis, stage, and T classification, while no significant difference was observed for other parameters such as age, gender, and grade (**Figure 2D**).

### The Prognostic Signature-Based Risk Score Was an Independent Prognostic Factor in the TCGA OSCC Cohort

**Figure 3:**
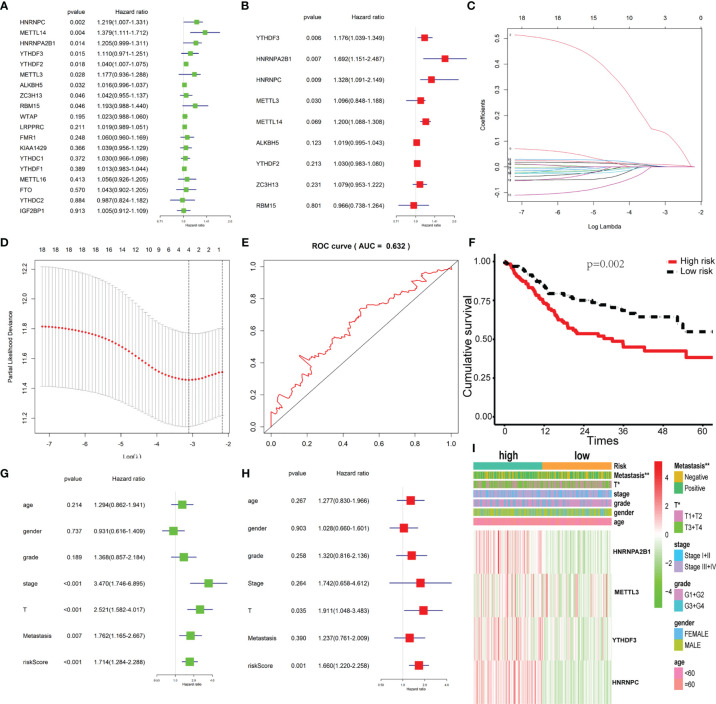
Construction of the Cox regression model and validation of the prognostic signature. **(A, B)** Univariate and multivariate cox regression based on 19 m^6^A-related genes. **(C)** LASSO coefficient profiles of 19 m^6^A-related genes associated with the overall survival of OSCC; each curve represents a gene. **(D)** Partial likelihood deviance is shown against log (lambda). The vertical dotted line indicated the lambda value with the minimum error and the largest lambda value, where the deviance is within one SE of the minimum. **(E)** The prognostic signature model showed good prediction efficiency with the area under the ROC curve (AUC = 0.632). **(F)** Survival analysis based on risk score. **(G, H)** Univariate cox regression and multivariate cox regression according to risk score and clinical characteristics. **(I)** The heatmap of risk score level and clinical characteristics. * and ** indicated p<0.05 and p<0.01, respectively.

Univariate and multivariate Cox regression analyses were used to identify the m^6^A-related genes that are associated with OS in the TCGA OSCC cohort. The results show that METTL3, YTHDF3, HNRNPC, and HNRNPA2B1 might be independent biomarkers in OSCC (**Figures 3A, B** **)**. Furthermore, LASSO Cox regression was performed to determine variables (**Figures 3C, D** **)**. Finally, four variables including METTL3, YTHDF3, HNRNPC, and HNRNPA2B1 were selected in Cox regression. The risk score for each patient was calculated with the following formula: risk scores = (0.1378*HNRNPA2B1) + (0.0036*METTL3) + (0.0021*YTHDF3) + (0.0095*HNRNPC). Then, all patients were grouped into the high-risk group and low-risk group, respectively. The survival analysis showed that the OSCC patients in the high-risk group had a significantly shorter overall survival than those in the low-risk group (**Figure 3F**). The prognostic signature model showed good prediction efficiency with the area under the ROC curve (AUC) value equal to 0.632 (**Figure 3E**). There were significant differences between the high-risk and low-risk groups with respect to the metastasis and T classification (**Figure 3I**). Here, we found that risk score may be an independent prognostic risk factor for OSCC (**Figures 3G, H** **)**.

### HNRNPA2B1 Expression Is Upregulated in OSCC

**Figure 4:**
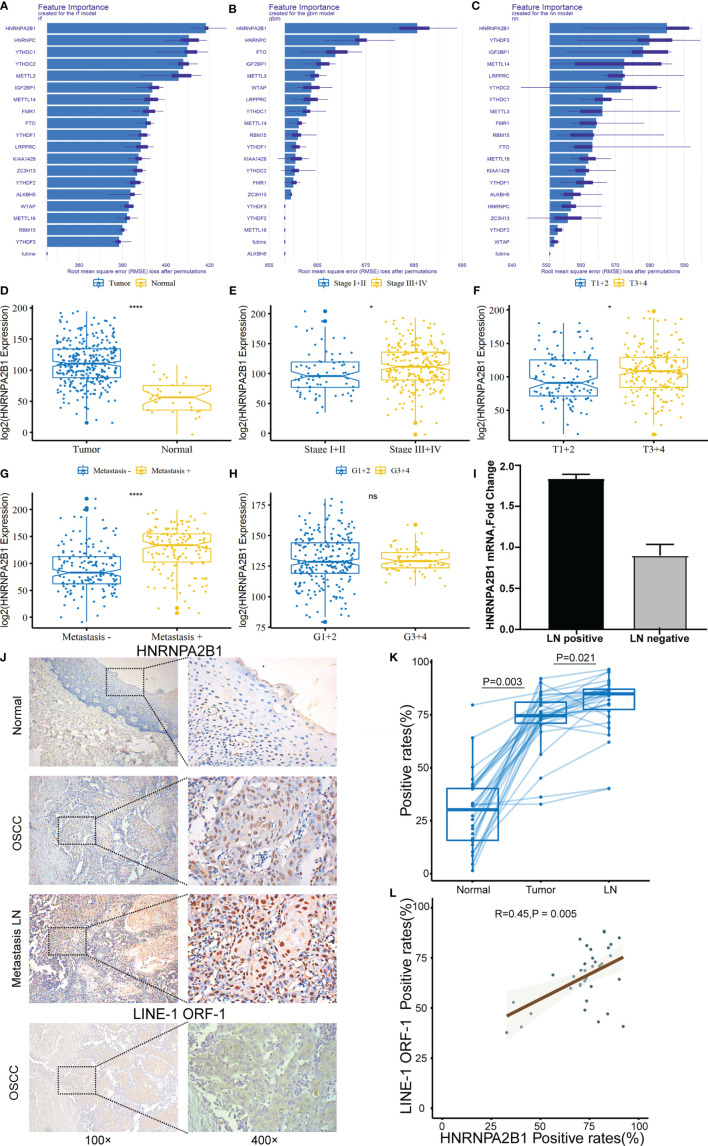
The correlation between HNRNPA2B1 and clinicopathologic parameters of OSCC. **(A–C)** Rank chart for the 19 m^6^A regulatory genes by neural network, random forest (rf), and gradient boosting machine (gbm). **(D)** HNRNPA2B1 expression is higher in tumor tissues compared with normal tissues. High HNRNPA2B1 expression was significantly associated with **(E)** tumor stage, **(F)** T categories, and **(G)** metastasis. **(H)** HNRNPA2B1 expression was not associated with histological grade (p > 0.05). **(I)** The mRNA expression of HNRNPA2B1 in patients with or without lymph node metastasis was detected by RT-PCR. **(J)** Representative images of immunohistochemical staining and **(K)** quantification of HNRNPA2B1 in human normal mucosa, OSCC tissue, and metastasis LN. Metastasis means at least one lymph node metastasis, local or distant metastasis. **(L)** LINE-1 expression is positively correlated with HNRNPA2B1 expression according to the analysis results of the IHC score. * and **** indicated p<0.05 and p<0.0001, respectively. “ns” indicates no significance.

To evaluate the important characteristics of the 19 m^6^A-related genes, we used the neural network (nn), random forest (rf), and gradient boosting machine (gbm) to build predictive models, respectively (**Figures 4A–C**). We found that the HNRNPA2B1 gene was consistently ranked the highest among the 19 genes in the three models, and it was selected as the most prognostically important locus of the 19 m^6^A regulatory genes in OSCC. Combining with the results of the previous univariate and multivariate Cox regression analyses, HNRNPA2B1 was selected to conduct further research. After analyzing the relationship between HNRNPA2B1 and each clinical parameter from the TCGA database, we found that HNRNPA2B1 was overexpressed in tumors (**Figure 4D**) and HNRNPA2B1 expression was significantly correlated with tumor stage, T-class, and metastasis (**Figures 4E–G**) but without grade (**Figure 4H**). Moreover, the mRNA expression of HNRNPA2B1 was examined in eight patients’ fresh cervical lymph nodes samples (four lymph node metastasis and four non-lymph node metastasis) by real-time PCR. The results showed that HNRNPA2B1 mRNA levels in lymph node metastasis patients were generally much higher than those in non-lymph node metastasis patients (the mean CT values of tumor samples with or without lymph node metastasis were 15.2572 and 16.5325, respectively. The mean ΔCT values were -0.675 and 0.1675, respectively, and the mean fold changes were 1.794 and 0.829, respectively) (**Figure 4I**). Moreover, to evaluate the expression of HNRNPA2B1 in OSCC, we examined HNRNPA2B1 expression of 38 OSCC patient tissues from our department by immunohistochemistry (IHC) staining. The expression status of HNRNPA2B1 was observed mainly in the nucleus of cells. After the IHC was scored, the results indicated lower HNRNPA2B1 expression in normal cases and higher expression in tumor tissues and metastatic lymph node (**Figures 4J, K** **)**. These results were consistent with the HNRNPA2B1 mRNA expression results from the TCGA database (**Figure 4D**). Fold changes were determined by comparing the ΔCT value of each product normalized to GAPDH as an internal control.

### Increased HNRNPA2B1 Expression Is Associated With OSCC Progression

**Figure 5:**
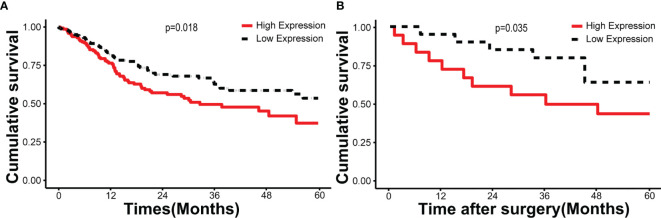
HNRNPA2B1 is associated with prognosis in patients with OSCC. Kaplan–Meier survival of **(A)** 330 cases from the TCGA database and **(B)** 38 patients from our department.

According to the follow-up data of the 38 OSCC patients from our department, we found that patients with high HNRNPA2B1 expression had a significantly poor 5-year OS compared with those with low expression of HNRNPA2B1 (p < 0.05, **Figure 5A**). The survival analysis from TCGA further validated this result (p < 0.05, **Figure 5B**). The univariate and multivariate Cox proportional hazards regression analyses revealed that HNRNPA2B1 expression was an independent prognostic factor for poor OS (p < 0.001, **Table 1**). Interestingly, the relationship between HNRNPA2B1 expression and the clinicopathological characteristics was statistically analyzed, and the results showed a direct association between HNRNPA2B1 expression and histological grade and metastasis (**Table 2**). Here, the definition of smoking is as follows: participants who smoked more than 100 cigarettes were classified as smokers (36). The definition of drinking is as follows: the participant had drunk alcohol regularly (i.e., drank at least once a week on a regular basis) during the past 12 months, otherwise were considered non-drinkers (37).

**Table 2:** Association between the clinicopathological characteristics and HNRNPA2B1 expression in 38 OSCC patients.

Variables	No.	HNRNPA2B1 expression		
		Negative (n)	Positive (n)	*p*
Gender				0.896
Male	29	9	20	
Female	9	3	6	
Age (years)				0.938
<60	25	8	17	
>=60	13	4	9	
Smoking				0.852
Yes	23	7	16	
No	15	5	10	
Drinking				0.825
Yes	20	6	14	
No	18	6	12	
T classification				0.031*
III–IV	16	2	14	
I–II	22	10	12	
Clinical stage				0.896
1+2	9	3	6	
3+4	29	9	20	
Histological grade				0.036*
Poor	12	1	11	
Well-moderate	26	11	15	
Metastasis				
+	24	4	20	0.01**
-	14	8	6	

Metastasis refers to at least one lymph node metastasis or local or distant metastasis; * and ** indicated p<0.05 and p<0.01, respectively.

### HNRNPA2B1 Expression Was Positively Related With the Proliferation, Migration, and Invasion of OSCC Cells *In Vitro*

**Figure 6:**
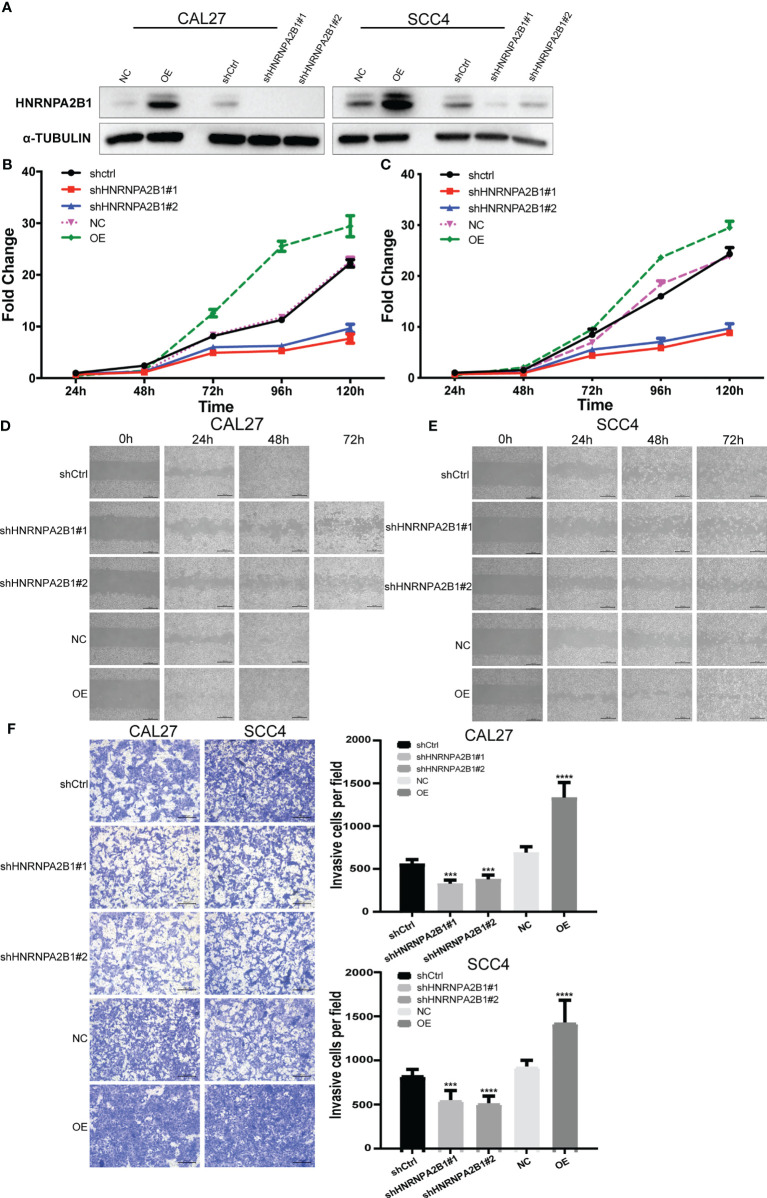
The expression of HNRNPA2B1 significantly affected the proliferation, migration, and invasion in OSCC cell lines. **(A)** Western blot was used to detect the protein expression after stable knockdown or overexpress HNRNPA2B1 in CAL27 and SCC4 cell lines. **(B, C)** Knockdown of HNRNPA2B1 inhibited cell proliferation as indicated by CCK-8 assay in CAL27 and SCC4 cells. On the contrary, overexpression of HNRNPA2B1 could enhance its proliferation. **(D, E)** Wound healing assay showed that downregulation of HNRNPA2B1 inhibits cell migration while HNRNPA2B1 overexpression could increase cell migration in CAL27 and SCC4. **(F)** Trans-well assay showed that the invasion abilities of CAL27 and SCC4 cells were impaired after knocking down HNRNPA2B1 and were increased after overexpression of HNRNPA2B1 compared with those of the control group. The left panel shows the representative images. The right panel shows the statistic data of the left panel. The data are presented as the mean of three independent experiments. *** and **** indicated p<0.001 and p<0.0001, respectively.

CAL27 cell and SCC4 cell were selected to establish HNRNPA2B1 knockdown and overexpress stable cell lines by lentiviral vectors. Western blot was used to measure the knockdown efficacy (**Figure 6A**).

The CCK8 assay indicated that downregulation of HNRNPA2B1 significantly inhibited cell growth compared with the control (**p < 0.01) (**Figures 6B, C** **)**. Wound healing assay was performed to determine the role of HNRNPA2B1 in regulating OSCC cell migration. After knocking down HNRNPA2B1#1 and HNRNPA2B1#2, the cell migration ability was remarkably suppressed (**Figures 6D, E** **)**. In addition, Transwell assay showed a significant decrease in the HNRNPA2B1 knockdown group (**Figure 6F**). We also found that overexpression of HNRNPA2B1 could enhance the proliferation, migration, and invasion ability of OSCC cells (**Figures 6B–F**). Together, we found that HNRNPA2B1 was positively correlated with OSCC progression.

### Altered Expression of HNRNPA2B1 Induces Epithelial and Mesenchymal Transition Changes

**Figure 7:**
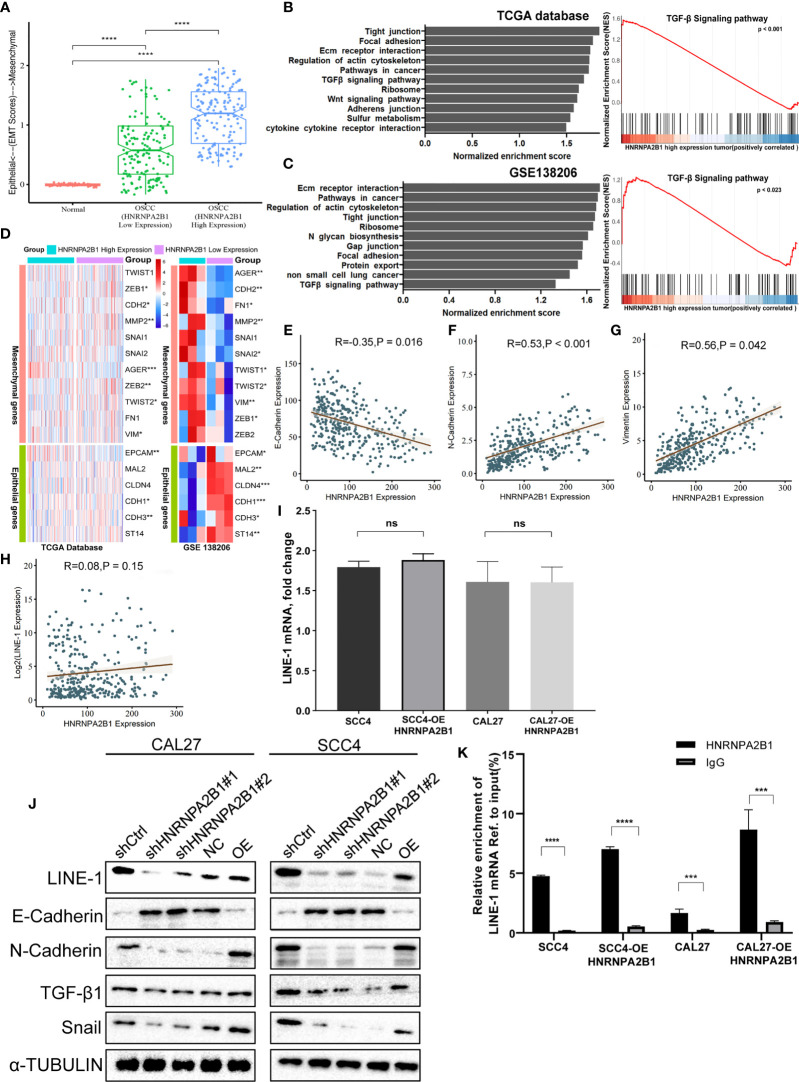
HNRNPA2B1 is correlated with the EMT process in OSCC. **(A)** EMT scores in normal and OSCC tissues. **(B, C)** Gene set enrichment analyses show the upregulated genes for HNRNA2B1 high-expression patients compared to low-expression patients. **(D)** GSE data (GSE138206) and TCGA data show that HNRNPA2B1 is correlated with multiple EMT markers. **(E–H)** HNRNPA2B1 expression is negatively correlated with E-cadherin (R = -0.35, p < 0.05) expression and positively correlated with N-cadherin (R = 0.53, p < 0.05) and vimentin (R = 0.56, p < 0.05), but was not correlated with the LINE-1 mRNA expression (R = 0.08, p = 0.15). **(I)** LINE-1 mRNA expression was not correlated with the HNRNPA2B1 expression in OSCC cell lines. **(J)** Western blot analysis of EMT markers related with LINE-1/TGF-β1/Snail signaling pathway. Protein levels of N-cadherin, E-cadherin, TGF-β1, Snail, and LINE-1 ORF1 of CAL27 and SCC4 cells were detected by Western blot after HNRNPA2B1 was knocked down or overexpressed. **(K)** Relative enrichment of LINE-1 mRNA associated with HNRNPA2B1 protein was identified by RIP assays using anti-IgG and anti-HNRNPA2B1 antibodies. The IgG group was a negative control to preclude nonspecific binding. The Y-axis represents the percent of input for each IP sample according to the formula: %Input =1/10*2^Ct [IP]-Ct [input]^. *, **, *** and **** indicated p<0.05, p<0.01, p<0.001 and p<0.0001, respectively. “ns” indicates no significance.

As a tumor-related gene, HNRNPA2B1 is associated with tumor progression (30). To investigate the mechanisms, we divided the OSCC patients into two groups (HNRNPA2B1 low expression group and HNRNPA2B1 high expression group). Through GSEA, we found that the top upregulated gene sets in the HNRNPA2B1 high expression group were related to EMT, such as tight junction, focal adhesion, adherens junction, and TGF-β signaling pathway (**Figures 7B, C** **)**. Moreover, the HNRNPA2B1 high expression group had a higher EMT score (**Figure 7A**) and multiple EMT markers were differential expression in the HNRNPA2B1 high and low expression group (**Figures 7D–G**). Epithelial–mesenchymal transition (EMT) is always observed at invasive OSCC and is significantly correlated with metastasis in tumor progression (38). In this study, we showed that reduced expression of HNRNPA2B1 in OSCC cells leads to decreased proliferation, migration, and invasion. These results illuminate that HNRNPA2B1 may promote OSCC progression by regulates EMT.

### HNRNPA2B1 May Regulate the Proliferation, Migration, and Invasion of OSCC Cells by Targeting EMT *via* the LINE-1/TGF-β1/Snail Signaling Pathway

The TGF-β signaling pathway is a classic pathway that promotes EMT (39), GSEA indicated that the TGF-β signaling pathway in the HNRNPA2B1 high expression group was stimulated (**Figures 7B, C** **)**. Our previous research demonstrated that LINE-1 may mediate the EMT process through the TGF-β1/Smad2/Snail signaling pathway in OSCC. HNRNPA2B1, as an RNA-binding protein, participates in the translation of many proteins (40–42). To explore whether HNRNPA2B1 plays a role in LINE-1 expression and whether HNRNPA2B1 mediates the EMT process by regulating the expression of LINE-1, we initially analyzed the transcription and translation of LINE-1 and HNRNPA2B1. The mRNA expression profile data of HNRNPA2B1 and LINE-1 from the TCGA database and show that their mRNA expression levels were not correlated (**Figure 7H**). Through the immunohistochemistry results of patient tumor tissues of 38 OSCC patients, we found that the protein levels of HNRNPA2B1 and LINE-1 are significantly positively correlated (**Figure 4L**).

Then, we examined the transcription and translation of LINE-1 upon HNRNPA2B1 loss. As expected, HNRNPA2B1 overexpression increased the protein abundance of LINE-1 but did not affect its RNA level in both CAL27 and SCC4 cells (**Figures 7I, J** **)**. Furthermore, we detected the EMT-related markers as indicated by Western blot after downregulating and upregulating the expression of HNRNPA2B1 in CAL27 and SCC4 cells (**Figure 7J**). The results were consistent with the results from TCGA.

To find out the precise mechanisms underpinning the observed HNRNPA2B1-dependent phenotypes, we conducted RIP assay using the HNRNPA2B1 antibody in OSCC cells to identify HNRNPA2B1 as m^6^A “reader” to combine with LINE-1 mRNA and promote the translation of LINE-1. We found that HNRNPA2B1 could enrich LINE-1 mRNA, implying that LINE-1 may be regulated in protein level upon interaction with HNRNPA2B1 (**Figure 7K**).

These results explain that HNRNPA2B1 may, as an m^6^A “reader,” regulate the proliferation, migration, and invasion of OSCC cells by targeting EMT *via* the LINE-1/TGF-β1/Sail signaling pathway.

## Discussion

m^6^A modification plays important roles in the eukaryotic RNA metabolism which is closely related to the initiation and progression of many types of tumors (43). However, at present, the role of m^6^A modification in the occurrence and development of OSCC is unclear. In this study, we demonstrated that m^6^A modifications dysregulated in OSCC tissues. HNRNPA2B1, as an m^6^A component, is overexpressed in OSCC and is a promising independent prognostic risk factor for OSCC. Furthermore, functional studies suggested that HNRNPA2B1 may promote the proliferation, migration, and invasion of OSCC cells by targeting EMT *via* the LINE-1/TGF-β1/Snail signaling pathway.

In recent years, more and more m^6^A components have been identified. In this study, we found that the m^6^A level in OSCC was upregulated, and 13 m^6^A genes were differentially expressed (shown in **Figure 1**). In addition, according to m^6^A-related gene expression, we divided the patients into cluster 1 and cluster 2 groups. The results showed that there was a significant difference in OS between the two groups (shown in **Figure 2**), which indicates that m^6^A dysregulation may contribute to the occurrence and development of OSCC. Based on univariate and multivariate Cox regression analyses, we found that METTL3, HNRNPA2B1, YTHDF3, and HNRNPC are four independent prognostic risk factors for OSCC. Subsequently, we established a Cox regression model; the patients were divided into high-risk and low-risk groups. The results show that in the high-risk group, the OS was poor, and the risk score may be related to the T classification and metastasis, but not related to other clinical parameters (shown in **Figure 3**). However, a similar study on head and neck squamous cell carcinoma (HNSCC) showed that HNRNPC and YTHDC2 are independent prognostic factors for HNSCC, and the risk score is related to age, gender, stage, and grade (44). Another similar study for OSCC indicated that HNRNPC, METTL14, YTHDF2, and ALKBH5 are independent prognostic factors for OSCC (45). The different results may be due to the inconsistent tumor locations and inclusion of more m^6^A genes in our study. Our present study indicates that HNRNPA2B1 may be a “key gene” involved in the process of m^6^A regulation disorder, leading to the occurrence and development of OSCC (shown in **Figures 4A–C**).

HNRNPA2B1 is a pre-mRNA-binding protein that participates in mRNA subcellular localization, stability, and translation (28). The latest research shows that it is the m^6^A “reader” that recognizes specific m^6^A motifs on mRNA, then combines it and regulates its expression (43). As early as 1999, scholars studied the tissue samples of seven OSCC patients through IHC and found that HNRNPA2B1 is highly expressed in OSCC tissues (46). Unfortunately, so far, the molecular mechanism of HNRNPA2B1 in OSCC is still unclear. In this study, we found that HNRNPA2B1 expression was correlated with patient survival, clinical stage, T classification, and lymph node metastasis (shown in **Figures 4D–K** and **Tables 1**, **2**). The correlation between HNRNPA2B1 and lymph node metastasis was further confirmed by the RT-PCR analysis of eight patients’ fresh cervical lymph nodes samples (shown in **Figure 5C**). It is said that more than 50% of OSCC patients have detectable lymph node metastasis, and patients with lymph node metastasis have a markedly worse prognosis than patients without metastasis (47). All above findings indicated that HNRNPA2B1 may have prognostic relevance with OSCC. Interestingly, regarding the relationship between HNRNPA2B1 and histological type, the result from TCGA (*p* > 0.05) was not consistent with our clinical result (*p* = 0.021*). This difference may be due to our insufficient patient samples. Hence, in the present study, besides the data from the TCGA database, we focused on the expression of HNRNPA2B1 in tissues or cell lines. The results showed that HNRNPA2B1 expression was higher in OSCC patient tissues than control (shown in **Figures 4J, K** **)**.

To examine whether HNRNPA2B1 is causatively involved in OSCC development, we downregulated the expression of HNRNPA2B1. We found that knockdown of HNRNPA2B1 in CAL27 and SCC4 cells inhibited their proliferation, migration, and invasion (shown in **Figures 6B–F**). Moreover, upregulation of HNRNPA2B1 was found to enhance the proliferation, migration, and invasion ability of OSCC cells (**Figures 6B–F**). These data suggest that HNRNPA2B1 is an important factor participating in the progression of OSCC. GSEA results show that the top upregulated gene sets in the HNRNPA2B1 high expression group were related to EMT, such as tight junction, focal adhesion, adherens junction, and TGF-β signaling pathway (**Figures 7B, C** **)**. Moreover, the HNRNPA2B1 high expression group had higher EMT scores (**Figure 7A**) and multiple EMT markers were differentially expressed in the HNRNPA2B1 high and low expression group (**Figures 7D–G**). These results were consistent with previous studies that HNRNPA2B1 promotes tumor progress through regulating EMT in colorectal cancer (CRC) (42), breast cancer (48), and pancreatic cancer (49). The role of EMT in regulating cancer processes such as initiation, proliferation, migration, and metastasis has been extensively investigated (50) in almost all cancers with sophisticated mechanisms. EMT status can be determined by the expression of specific markers. CDH1 (E-cadherin) expressions always served as tumor suppressors during carcinoma EMT (51), while the hallmarks of EMT are the upregulation of CDH2 (N-cadherin) and vimentin (52). Our previous study indicated that LINE-1 is correlated with EMT progression in OSCC (not shown). Here, in the present study, we found that LINE-1 expression was decreased, and E-cadherin and N-cadherin were abnormally expressed after depletion of HNRNPA2B1 (**Figure 7G**). However, after upregulating the expression of HNRNPA2B1, we got the opposite result (**Figure 7G**), which might explain the cell growth inhibition and lower relative invasiveness after HNRNPA2B1 was reduced. Our previous research indicates that LINE-1 regulates the proliferation and migration of OSCC cells by targeting EMT *via* the TGF-β1/Smad2/Snail signaling pathway. We found that Snail and TGF-β1 expression was suppressed after silencing of HNRNPA2B1 (**Figure 7G**). In this study, we also found that overexpression of HNRNPA2B1 increased the protein abundance of LINE-1 but did not affect its RNA level in OSCC cells. Further, through RIP assay we found that HNRNPA2B1 could enrich LINE-1 mRNA, implying that LINE-1 may be regulated in protein level upon interaction with HNRNPA2B1. The results were consistent with previous studies that HNRNPA2B1 acts as an m^6^A reader, binding and affecting m^6^A methylated transcripts, and then regulating the translation initiation, splicing, exportation, and other processes of the target gene to perform its functions (53–57). These results suggested that HNRNPA2B1 as an m^6^A reader may regulate the development of OSCC by targeting EMT *via* the LINE-1/TGF-β1/Snail signaling pathway. However, more work is needed for further studies, such as whether HNRNPA2B1 recognizes the m^6^A motif in LINE-1 mRNA and regulates its translation to mediate the EMT process.

Taken together, HNRNPA2B1, as an m^6^A reader, is critical in OSCC development. Its expression is significantly associated with the prognosis of OSCC. Moreover, m^6^A may act as a proto-oncogene that promotes the OSCC proliferation, migration, and invasion through the EMT progression *via* the LINE-1/TGF-β1/Snail signaling pathway. These findings may provide a new strategy for OSCC therapy.

## Data Availability Statement

The original contributions presented in the study are included in the article/supplementary material. Further inquiries can be directed to the corresponding author.

## Ethics Statement

The studies involving human participants were reviewed and approved by the Ethics Committee of the Second Xiangya Hospital of Central South University, Changsha, China. The patients/participants provided their written informed consent to participate in this study.

## Author Contributions

CF and FZ designed the experiments. TY, TS, MY, and FZ performed the experiments and analyzed the data. FZ and TY wrote the article. CF and FZ modified the manuscript. All authors contributed to the article and approved the submitted version.

## Conflict of Interest

The authors declare that the research was conducted in the absence of any commercial or financial relationships that could be construed as a potential conflict of interest.

## Publisher’s Note

All claims expressed in this article are solely those of the authors and do not necessarily represent those of their affiliated organizations, or those of the publisher, the editors and the reviewers. Any product that may be evaluated in this article, or claim that may be made by its manufacturer, is not guaranteed or endorsed by the publisher.

## Abbreviations

m^6^A, N6-methyladenosine; OSCC, oral squamous cell carcinoma; EMT, epithelial and mesenchymal transition; mRNA, messenger RNA; HNRNPA2B1, heterogeneous nuclear ribonucleoproteins A2B1; LINE-1, long interspersed nucleotide element 1; TCGA, The Cancer Genome Atlas; AUC, area under the ROC curve; IHC, immunohistochemistry.
